# 

**Published:** 1892-10

**Authors:** 


### CONTENTS.

ORIGINAL COMMUNICATIONS—

The Relations of Bacteria to Disease. By Charles M. Blackford, M. D., Lynchburg, Va.... 449
Abdominal Hysterectomy for Large Fibroids, with Report of Case. By B. W. Bizzell, M. D., Atlanta, Ga.... 457
Outline of the History of Malignant or Asiatic Cholera in New Orleans, La. By Joseph Jones, M. D., LL. D., New Orleans, La.... 465
Supra-Pubic Cystotomy, with Removal of Oxalate of Lime Stone. By Jas. S. White, M. D., Columbia, Ky.... 469

SOCIETY REPORT—

Allegheny County Medical Society.... 471

CORRESPONDENCE—

From Paris.... 477
From New York.... 480

EDITORIAL—

To Our Subscribers.... 485
George P. Rowell and Company, Publishers of the American Newspaper Directory.... 486
The Dose of Nitroglycerine.... 487
The Treatment of Typhoid Fever by Intestinal Antiseptics.... 488
The Ideal Physician.... 489

SELECTIONS.... 491
MEDICAL ITEMS.... 505
BOOK REVIEWS.... 509
INDEX TO ADVERTISEMENTS.... xi
ITEMS OF INTEREST.... xii, xxix-xxxii

